# Development and application of a quadruple RT-qPCR assay for the simultaneous detection of NoV GI, NoV GII, and HAV in bivalve shellfish

**DOI:** 10.1128/aem.01839-24

**Published:** 2024-12-19

**Authors:** Yan Wang, Jinfeng Wang, Maolin Wei, Libing Liu, Jianchang Wang, Xiangdong Xu

**Affiliations:** 1 School of Public Health, Hebei Medical University 580290 , Shijiazhuang, China; 2 Food Microbiology and Animal Quarantine Laboratory, Technology Center of Shijiazhuang Customs, Shijiazhuang, China; 3 Hebei Key Laboratory of Environment and Human Health, Shijiazhuang, China; The Pennsylvania State University, University Park, Pennsylvania, USA

**Keywords:** noroviruses, hepatitis A virus, quadruple RT-qPCR, simultaneous detection, bivalve shellfish, MS2, process control virus

## Abstract

To achieve rapid and simultaneous detection of NoV GI, NoV GII, and HAV, a quadruple real-time fluorescence quantitative PCR (RT-qPCR) assay was developed using MS2 bacteriophage as a process control virus. The quadruple RT-qPCR assay effectively detected NoV GI, NoV GII, HAV, and MS2 RNA with detection limits of 10^2^ copies/μL, 10^3^ copies/μL, 10^2^ copies/μL, and 10^3^ copies/μL, respectively, within 1 hour 50 minutes. The quadruple RT-qPCR assay could specifically detect NoV GI, NoV GII, HAV, and MS2 without cross-reactions with other common pathogens, demonstrating good reproducibility with intra-assay and inter-assay coefficients of variation all below 2.11%. In this study, 337 bivalve shellfish samples collected from various regions of Hebei Province were pretreated using the proteinase K-PEG 8000 precipitation-chloroform method, and viral nucleic acids were enriched and extracted from a volume of viral solution that was doubled. The developed quadruple RT-qPCR assay was used to detect NoV GI, NoV GII, and HAV in bivalve shellfish samples, and the positive rates were 19.88% (67/337), 20.47% (69/337), and 4.75% (16/337), respectively. In addition, mixed infections of NoV GI and NoV GII (10.68%, 36/337) and NoV GI and HAV (0.89%, 3/337) were observed. In all, 200 bivalve shellfish samples were randomly selected for the assay, and it was found that the total, positive, negative coincidence rates, and Kappa values of the quadruple RT-qPCR assay were 98.3%, 99.1%, 98.2%, and 0.945, respectively, compared with the single RT-qPCR assay. These results show that the developed quadruple RT-qPCR assay has comparable performance to the single RT-qPCR assay.

**IMPORTANCE:**

Food-borne diseases caused by viral contamination have become a growing concern, and bivalve shellfish is a crucial source of infection, with many outbreaks of non-bacterial acute gastroenteritis associated with raw food or the use of undercooked shellfish such as oysters. As food contamination problems caused by NoV and HAV become more severe, it is important to study and establish a sensitive and efficient assay to simultaneously detect NoV and HAV by applying the MS2 process control virus for the protection of bivalve shellfish food safety and the monitoring of the above food-borne viral contamination. In addition, bivalve shellfish samples contain a large number of PCR inhibitors such as polysaccharides, lipids, and proteins, so optimization of the virus enrichment and extraction method is essential and is expected to provide a research basis for subsequent related experiments.

## INTRODUCTION

Food-borne diseases caused by viral contamination have become a growing concern (1, 2). Recent studies indicate that over 50% of viral food-borne diseases globally manifest as non-bacterial acute gastroenteritis, presenting symptoms such as nausea, vomiting, abdominal pain, low-grade fever, headache, muscle pain, fatigue, and loss of appetite, commonly referred to as gastrointestinal influenza (3). These illnesses affect all age groups, with children being particularly vulnerable (4). In recent years, the prevalence of non-bacterial gastroenteritis caused by norovirus (NoV) in China has been increasing annually. NoV was reported to be the second most important cause of human virus acute gastroenteritis in children less than 5 years of age worldwide, following rotavirus (5). Non-bacterial acute gastroenteritis can be caused by various pathogens, including NoV, hepatitis A virus (HAV), rotavirus (RV), human astrovirus (HAstV), sapovirus (SaV), enteroviruses (EV), and adenovirus (AdV) (6). Moreover, food-borne viral contamination often involves multiple viruses, leading to mixed infections that pose severe threats to food safety and public health, and complicate the detection and identification of these viruses (7–9).

Hebei Province is located in northern China and has a long coastline, especially bordering the Bohai Sea, which makes the region easily accessible to a wide range of marine seafood products. In addition, the local population is fond of consuming bivalve shellfish. However, bivalve shellfish could be contaminated during transport, storage, and cooking, which poses a threat to people’s health. Food-borne viruses are primarily transmitted through the fecal-oral route (10), and the consumption of contaminated food or water, as well as person-to-person contact, is a significant mode of transmission for NoV and HAV outbreaks. Foods prone to food-borne viral infections include raw or undercooked meat or bivalve shellfish, fruits, vegetables, and convenience foods that lack adequate heat treatment (11, 12). Among these, bivalve shellfish are a crucial source of infection (13). As filter-feeding organisms, bivalve shellfish accumulate and concentrate viruses from contaminated water within their digestive tissues. Through this process, bivalve shellfish can concentrate viruses up to 99 times more than the surrounding water (14), making them significant vectors for the transmission of food-borne pathogens (15). Most bivalve shellfish are consumed raw or minimally cooked, often steamed for only a few minutes (16). Due to the high survival rate of food-borne pathogens, their high infectivity, and low infectious dose, sometimes as few as 10–100 virus particles can cause an infection (17, 18). Epidemiological investigations and laboratory validations have shown that many acute gastroenteritis outbreaks are associated with the consumption of raw or undercooked oysters and other bivalve shellfish (19, 20). In addition, the complex matrix of bivalve shellfish samples and their low viral load (21) means that PCR inhibitors such as proteins and lipids present in bivalve shellfish can lead to false-negative results. Effective virus concentration, efficient nucleic acid extraction, and successful target gene amplification are critical for detecting food-borne viruses in bivalve shellfish. Therefore, optimizing the optimal virus concentration and extraction methods and developing sensitive and efficient methods for the detection of NoV and HAV in bivalve shellfish are important for tracing the source of food-borne disease outbreaks and ensuring food safety.

With the prevalence of food contamination problems caused by NoV and HAV, detection technology forms the foundation for monitoring, preventing, and controlling food-borne diseases. The quadruple RT-qPCR assay saves a lot of time and reagents compared to the single RT-qPCR assay. Although many multiplex RT-qPCR assays have been developed for the detection of food-borne viruses, these assays do not include process control viruses, which makes it impossible to also assess the validity of the detection process (22). In this study, a quadruple RT-qPCR assay based on TaqMan probes was developed for the simultaneous identification and detection of NoV GI, NoV GII, and HAV, and the effectiveness of the assay process was assessed by MS2 bacteriophage and corresponding EC RNA. The developed method was used to detect these food-borne viruses in commercially available bivalve shellfish from different regions of Hebei Province.

## MATERIALS AND METHODS

### Bacteria, viruses, and samples

*Salmonella* (CICC22956) and *Escherichia coli* (CICC10899) were obtained from the China Center of Industrial Culture Collection, while *Escherichia coli* (ATCC15597) was obtained from the American Type Culture Collection. MS2 bacteriophage (ATCC 15597-B1) was kindly provided by the Technology Center of Qingdao Customs. The nucleic acids of NoV GI, NoV GII, HAV, hepatitis E virus (HEV), HAstV, and SaV were preserved at the Technology Center of Shijiazhuang Customs.

A total of 337 bivalve shellfish samples, including clams, oysters, scallops, and razor clams, were collected from seafood markets, agricultural trade markets, and supermarkets across various regions of Hebei Province between May and December 2023. All samples were either immediately sent for testing or stored below −20°C until testing.

### Quadruple RT-qPCR primers and probes

According to ISO 15216-2 (2019) (23), primers and TaqMan probes targeting the ORF1/ORF2 junction of NoV GI and NoV GII were synthesized. Primers and TaqMan probes targeting the highly conserved region of the 5′ UTR of HAV were also synthesized. According to the literature (24), primers and TaqMan probes targeting the rep gene of MS2 were synthesized. The 5′ ends of the probes for NoV GI, NoV GII, HAV, and MS2 were labeled with VIC, CY5, FAM, and ROX fluorescent dyes, respectively, while the 3′ ends were labeled with BHQ1, BHQ1, NFQ-MGB, and BHQ1 quenchers, respectively. The above-mentioned primers and probes are listed in Table 1 and were synthesized by Jierui Biological Engineering (Shanghai, China).

**TABLE 1:** Primer and probe oligonucleotides used for quadruple RT-qPCR

Virus	Primer	Primer sequence (5′→3′)	Product length (bp)	GenBank accession number and position	Reference
NoV GI	QNIF4	CGC TGG ATG CGN TTC CAT	86	M87661 5291–5376	(23)
	NV1LCR	CCT TAG ACG CCA TCA TCA TTT AC			
	NVGG1p	VIC-TGG ACA GGA GAY CGC RAT CT-BHQ1			
NoV GII	QNIF2	ATG TTC AGR TGG ATG AGR TTC TCW GA	89	X86557 5012–5100	
	COG2R	TCG ACG CCA TCT TCA TTC ACA			
	QNIFs	CY5-AGC ACG TGG GAG GGC GAT CG-BHQ1			
HAV	HAV68	TCA CCG CCG TTT GCC TAG	173	M59809 68–240	
	HAV240	GGA GAG CCC TGG AAG AAA G			
	HAV150	FAM-CCT GAA CCT GCA GGA ATT AA-MGB			
MS2	MS2-TM2-F	GGC TGC TCG CGG ATA CCC	202	JF719743.1 3135–3336	(24)
	MS2-TM2-R	TGA GGC AAT GTG GGA ACC G			
	MS2-TM2	ROX-ACC TCG GGT TTC CGT CTT GCT CGT-BHQ1			

### Generation of genetic reference material

Using primers and probes listed in Table 1, we amplified the ORF1/ORF2 junction of NoV GI and NoV GII, the 5′ UTR region of HAV, and part of the rep gene of MS2, resulting in target fragments of 86 bp, 89 bp, 173 bp, and 202 bp, respectively. These fragments were inserted into the pGEM-T Easy vector (Promega, US) to construct recombinant plasmids. The recombinant plasmids were quantified with a NanoDrop 2000c (Thermo Fisher Scientific, US) and linearized using the NdeI enzyme (Takara Biochemical Technology, Dalian, China), followed by separation by 1.0% agarose gel electrophoresis. The linear gel bands were excised and purified using an agarose gel DNA recovery kit (SanshiBio, Hebei, China). The purified products were transcribed *in vitro* using the T7 RiboMAX Express Large-Scale RNA Production System kit (Promega, US). The transcription products were treated with RNase-free DNase I (Tiangen Biotech Co., Ltd., Beijing, China) to remove residual DNA and purified using an RNA Clean Kit (Tiangen Biotech Co., Ltd., Beijing, China). qPCR was then performed on the transcription products to ensure DNA contamination was less than 0.10%. The purified transcription products were quantified with a NanoDrop 2000c. The number of RNA copies was calculated using the formula: (6.02 × 10^23^) × [X (ng/μL)] ×10^−9^/(RNA length × 340) = Y (copies/μL), and aliquots were stored at −80°C for future use.

### Optimization of the quadruple RT-qPCR assay

*In vitro* transcribed RNAs of NoV GI, NoV GII, HAV, and MS2 were diluted to 10^6^ copies/μL and used as experimental templates to optimize the reaction system and conditions. Based on the pre-configured buffer mixture (5 × RT qPCR Master Mix and dNTP mixed at 20:1) and enzyme mixture (Taq Hot-Start DNA Polymerase, HI-MMLV Reverse Transcriptase, and RNase Inhibitor mixed at 23:4:3) (SanshiBio, Hebei, China), additional essential components were added to the qPCR amplification mixture. Further optimization was conducted through matrix experiments on the concentrations of Mg^2+^ (MgCl_2_, 25 mM, SanshiBio, Hebei, China) with final concentrations of 0.5 × 10^3^ , 1.0 × 10^3^ , 1.5 × 10^3^ , 2.0 × 10^3^ µmol/L; dNTP with final concentrations of 1.0 × 10^3^ , 1.4 × 10^3^ , 1.8 × 10^3^ , 2.2 × 10^3^ , 2.6 × 10^3^ , 3.0 × 10^3^ µmol/L; Taq Hot-Start DNA Polymerase with final concentrations of 0.14 , 0.16 , 0.18 , 0.20 , 0.22 , 0.24 U/µL; primers with final concentrations of 0.60, 0.70, 0.80, 1.00, 1.20, 1.50 µmol/L; and probes with final concentrations of 0.30, 0.35, 0.40, 0.50, 0.60, 0.75 µmol/L. A 25 µL reaction system for quadruple RT-qPCR was established, with 2 µL of the template mixture (mixed at a 1:1:1:1 vol ratio) and ddH_2_O added to a total volume of 25 µL. After thorough mixing and brief centrifugation, amplification was performed using the Quant Studio 5 fluorescence PCR instrument (ABI, US) under the following conditions: 55°C for 10 minutes; 95°C for 10 minutes; 95°C for 15 s, 60°C for 30 s, 65°C for 30 s for 45 cycles, with fluorescence collected at 65°C. Conditions with the lowest Ct values, highest fluorescence intensity, smoothest amplification curves, and no non-specific amplification were selected as the optimal conditions. After determining the optimal reaction system, annealing temperatures were optimized at 53°C, 55°C, 57°C, 60°C, and 62°C.

### Specificity test of the quadruple RT-qPCR assay

The specificity of the quadruple RT-qPCR assay was verified using nucleic acids of NoV GI, NoV GII, HAV, and MS2, as well as nucleic acids of HEV, HAstV, SaV, *Escherichia coli*, and *Salmonella* genomic DNA and RNA, with ddH_2_O serving as a blank control. This specificity analysis was conducted in triplicate.

### Sensitivity test of the quadruple RT-qPCR assay

*In vitro* transcribed RNAs of NoV GI, NoV GII, HAV, and MS2 were serially diluted ten-fold to achieve concentrations ranging from 10^7^ to 10°Copies/μL. These diluted RNAs were mixed at a 1:1:1:1 vol ratio, and 2 µL of the mixture was used as the template to verify the sensitivity of the quadruple RT-qPCR assay. The sensitivity analysis was conducted in five replicates.

### Construction of standard curves for the quadruple RT-qPCR assay

*In vitro* transcribed RNA at concentrations ranging from 10^7^ to 10^3^ copies/μL was used as the template for the quadruple RT-qPCR detection. The Quant Studio 5 fluorescence PCR instrument software was utilized to perform the analysis and generate the standard curves.

### Reproducibility test of the quadruple RT-qPCR assay

*In vitro* transcribed RNA at three different concentrations (10^7^, 10^5^, and 10^3^ copies/μL) was used as templates for the quadruple RT-qPCR amplification. Each concentration was tested in triplicate to assess both intra-assay and inter-assay reproducibility. The coefficient of variation (CV) for intra-assay and inter-assay was calculated using statistical methods to evaluate the reproducibility of quadruple RT-qPCR assay.

### The single RT-qPCR assay

The single RT-qPCR assay was performed in a 25 µL volume using the One Step RT-PCR Kit (SanshiBio, Hebei, China). The reaction mixture contained 5 µL of 5 × One Step RT-qPCR Master Mix, 1 µL of One Step RT-qPCR Enzyme Mix, 1.0 µL of primers (10 µmol/L) and 0.5 µL of probe (10 µmol/L), 2 µL extracted nucleic acid, and 14.5 µl of ddH_2_O. The primers and probe were the same as the quadruple RT-qPCR assay (Table 1). The reaction conditions were 55°C for 10 minutes; 95°C for 10 minutes; 45 cycles of 95°C for 15 s, 55°C for 30 s, and 65°C for 30 s (fluorescence collected).

### MS2 bacteriophage titer determination

The MS2 bacteriophage suspension was serially diluted 10-fold. Each dilution was plated in triplicate and incubated for 17–24 hours following the method described in reference 25. The titer of the MS2 bacteriophage was determined by counting the plates with 30–300 plaques.

### Optimization of pretreatment methods for bivalve shellfish

Bivalve shellfish samples were processed according to ISO 15216-2 (2019). First, more than 10 bivalve shellfish were randomly selected from each batch of samples, the digestive glands were removed and placed in a sterile petri dish. Second, the digestive glands were roughly chopped using sterile scissors and 2.0 g was weighed in a 50 mL centrifuge tube. The 50 mL centrifuge tube with digestive glands was then placed in an ice box and the digestive glands were further ground using an electric mortar and pestle (Tiangen Biotech Co., Ltd., Beijing, China). To create simulated contaminated samples, 10 µL of MS2 bacteriophage at a titer of 10^11^ pfu/mL was added to the collected digestive glands and left to stand for 30 minutes to allow the virus to adsorb onto the tissue. Referring to ISO 15216-2 (2019) and the virus concentration methods described in the literature (26), three methods were used for processing: proteinase K treatment, proteinase K-Trizol lysis-chloroform extraction, and proteinase K-PEG 8000 precipitation-chloroform extraction. Nucleic acids were extracted from the treated samples and detected using the established quadruple RT-qPCR assay on an ABI Quant Studio 5 fluorescence PCR instrument. Each nucleic acid extraction method was repeated three times, with each repeat set in duplicate. Extraction efficiency was evaluated based on Ct values, recovery rates, and inhibition indices to determine the optimal viral nucleic acid extraction method. The recovery rate was calculated as (amount of recovered virus/amount of added virus × dilution factor) × 100%. The inhibition index was expressed as the difference in Ct values between the sample-extracted nucleic acid RNA containing process control viruses + 1 µL EC RNA and water +1 µL EC RNA. A recovery rate >1% and an inhibition index <2.00 (inhibition rate <75%) were considered acceptable.

#### Proteinase K method (Method 1)

Following ISO 15216-2 (2019), 1.0 mL of PBS solution and 10 µL of 20 mg/mL proteinase K (Tiangen Biotech Co., Ltd., Beijing, China) were added to the simulated contaminated sample and mixed well. The mixture was incubated at 37°C with shaking at 320 rpm for 60 minutes, followed by heating at 60°C in a water bath for 15 minutes. The sample was then centrifuged at 3,000 rpm for 5 minutes at room temperature, and the supernatant was collected. Viral RNA was extracted from the supernatant using the QIAamp Viral RNA Mini Kit (Qiagen, US) according to the manufacturer’s instructions. Unlike other research methods, in this study, when viral nucleic acids were extracted using the QIAamp Viral RNA Mini Kit, adsorption column filtration was performed on a double volume of supernatant to increase the concentrated viral loads.

#### Proteinase K-Trizol lysis-chloroform extraction method (Method 2)

Following and modifying ISO 15216-2 (2019), an equal volume of Trizol Reagent (Ambion, US) was added to the virus extract obtained from Method 1, mixed thoroughly, shaken vigorously, and allowed to stand at room temperature for 5 minutes. Then, 0.30 volumes of chloroform were added, mixed by hand inversion, and centrifuged at 12,000 rpm/min for 5 minutes. The upper aqueous phase was transferred to a new centrifuge tube. Viral RNA was extracted from the duplicate supernatant using the QIAamp Viral RNA Mini Kit according to the manufacturer’s instructions.

#### Proteinase K-PEG 8000 precipitation-chloroform extraction method (Method 3)

Add 1/4 vol of 5 × PEG8000/NaCl solution to the viral extract obtained in method 1 to precipitate the virus. The mixture was vortexed thoroughly and incubated at 4°C with shaking at 100 rpm/min for 60 minutes, followed by centrifugation at 12,000 rpm/min for 30 minutes at 4°C. The supernatant was discarded, and the pellet was resuspended thoroughly in 500 µL of PBS solution. An equal volume of chloroform-n-butanol solution (1:1 vol) was added to the PBS suspension for extraction. The mixture was vortexed thoroughly, incubated at room temperature for 10 minutes, and centrifuged at 12,000 rpm/min for 15 minutes at 4°C. The upper aqueous phase was transferred to a new centrifuge tube. Viral RNA was extracted from the duplicate supernatant using the QIAamp Viral RNA Mini Kit according to the manufacturer’s instructions.

### Detection of clinical samples

Following the optimal pretreatment method for bivalve shellfish samples determined in “Optimization of pretreatment methods for bivalve shellfish,” above, the established quadruple RT-qPCR assay was used to simultaneously detect NoV GI, NoV GII, and HAV in 337 collected bivalve shellfish samples of various types. MS2 bacteriophage served as a process control virus to calculate extraction efficiency, representing the recovery rate of food-borne viruses in the samples. *In vitro* transcribed RNA of the three food-borne viruses at 10^6^ copies/μL was used as external control RNA to calculate the amplification inhibition index and assess the method’s effectiveness. In all, 200 bivalve shellfish samples were randomly selected for single RT-qPCR detection of NoV GI, NoV GII, and HAV to compare the detection results of the two methods.

### Statistical analysis

Data entry and summary of shellfish product testing results were conducted using EXCEL 2021. Statistical analysis of the detection of NoV in bivalve shellfish products was performed using SPSS 25.0 software. The chi-square test was used to compare the detection rates of NoV and HAV in different bivalve species and the differences in detection rates between the quadruple RT-qPCR assay and the single RT-qPCR assay, with a significance level of α = 0.05. One-way ANOVA was used to compare the MS2 amplification Ct values and recovery rates obtained from three different pretreatment methods, also with a significance level of α = 0.05.

## RESULTS

### Optimization of the quadruple RT-qPCR

By optimizing key components, primer and probe concentrations, and annealing temperature, the optimized reaction system for quadruple RT-qPCR assay was established as follows: 5 µL of 5 × RT qPCR Master Mix, 0.25 µL of dNTP (final concentration 1.0 × 10^3^ µmol/L), 0.97 µL of Taq Hot-Start DNA Polymerase (final concentration 0.18 U/µL), 0.13 µL of HI-MMLV Reverse Transcriptase (final concentration 1.04 U/µL), 0.10 µL of RNase Inhibitor (final concentration 0.16 U/µL), 0.50 µL of MgCl_2_ (final concentration 5.0 × 10^2^ µmol/L), 1 µL each of forward and reverse primers (10 µmol/L) (final concentration 0.40 µmol/L), 0.75 µL of probes (10 µmol/L) (final concentration 0.30 µmol/L), and 2 µL of *in vitro* transcribed RNA for NoV GI, NoV GII, HAV, and MS2 mixed at a 1:1:1:1 vol ratio as the template, with ddH_2_O added to reach a final volume of 25 µL. The reaction conditions were 55°C for 10 minutes; 95°C for 10 minutes; 45 cycles of 95°C for 15 s, 55°C for 30 s, and 65°C for 30 s (fluorescence collected).

### Analysis of the specificity

**Fig 1:**
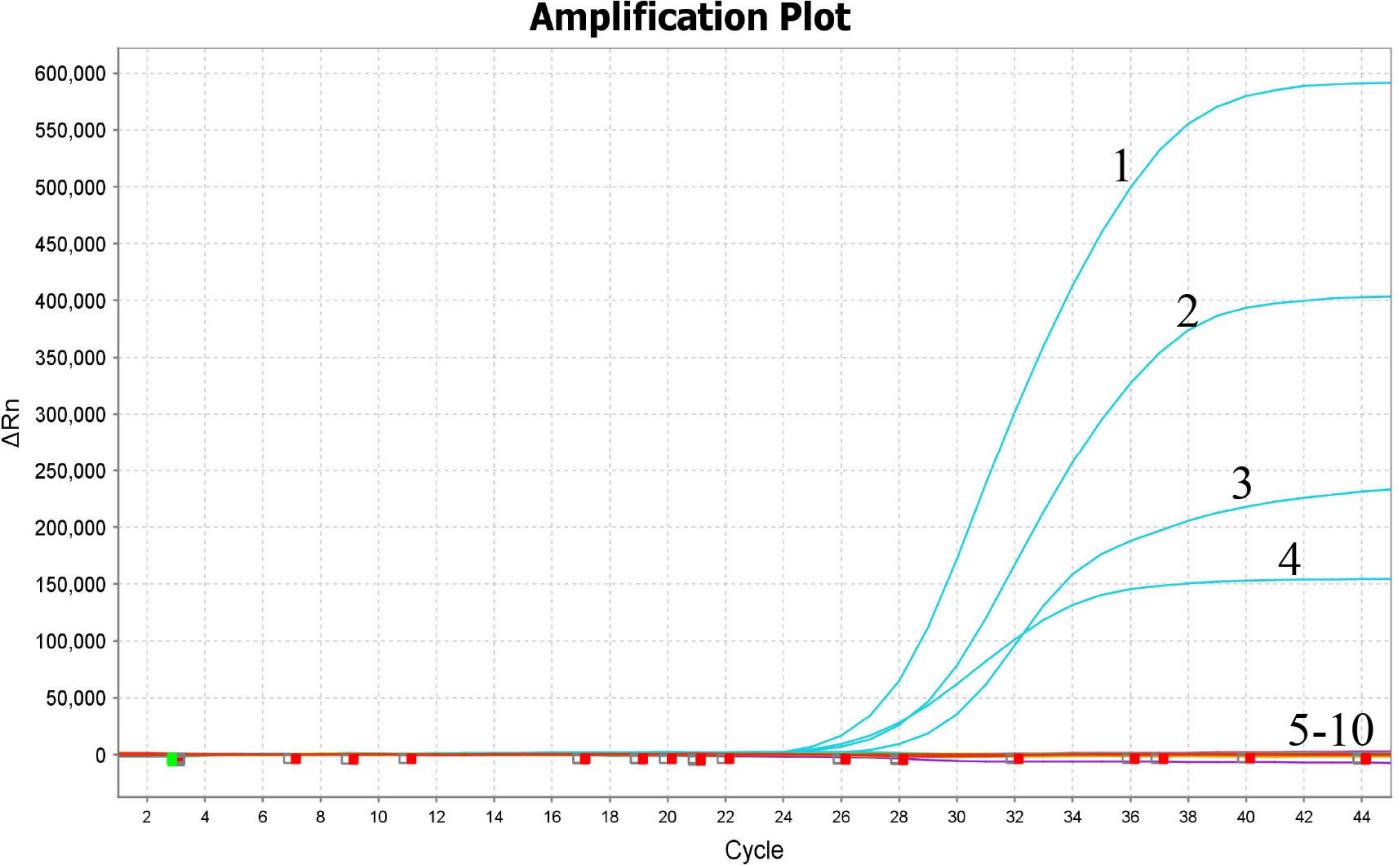
Specificity results of the quadruple RT-qPCR. Line 1, HAV; line 2, MS2; line 3, NoV GII; line 4, NoV GI; line 5, HEV; line 6, HAstV; line 7, SaV; line 8, *Escherichia coli*; line 9, *Salmonella*; line 10, ddH_2_O as no-template control.

The specificity of the quadruple RT-qPCR assay was validated using genomic RNA or DNA/cDNA from NoV GI, NoV GII, HAV, MS2, HEV, HAstV, SaV, *Escherichia coli*, and *Salmonella* as templates. The results showed that specific amplification curves were produced only for NoV GI, NoV GII, HAV, and MS2, with no amplification curves observed for other common food-borne pathogens within 45 cycles, indicating excellent specificity (Fig. 1). The results were consistent across three replicate experiments.

### Analysis of the sensitivity

**Fig 2:**
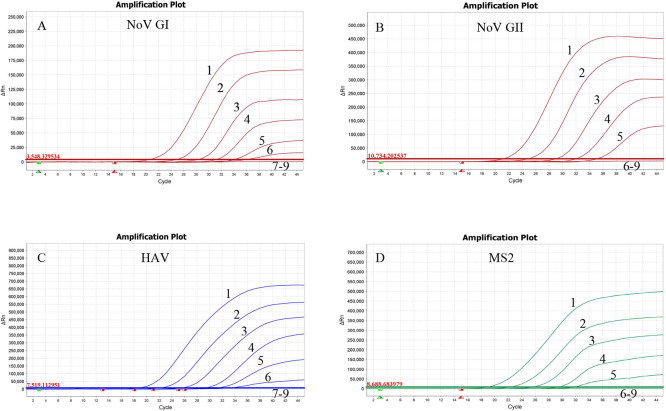
(A to D) Sensitivity results of NoV GI, NoV GII, HAV, and MS2 in the quadruple RT-qPCR. Line 1, 1 × 10^7^ copies/μL; line 2, 1 × 10^6^ copies/μL; line 3, 1 × 10^5^ copies/μL; line 4, 1 × 10^4^ copies/μL; line 5, 1 × 10^3^ copies/μL; line 6, 1 × 10^2^ copies/μL; line 7, 1 × 10^1^ copies/μL; line 8, 1 × 10°copies/μL; and line 9, ddH_2_O.

Ten-fold serial dilutions of *in vitro* transcribed RNA of NoV GI, NoV GII, HAV, and MS2 were used as templates, with 0.50 µL of each used to verify the sensitivity of the quadruple RT-qPCR assay. The results showed that NoV GI and HAV produced stable typical amplification curves at a template concentration of 10^2^ copies/μL, while NoV GII and MS2 produced stable typical amplification curves at a template concentration of 10^3^ copies/μL. Therefore, the method’s sensitivity is 10^2^ copies/μL for NoV GI, 10^3^ copies/μL for NoV GII, 10^2^ copies/μL for HAV, and 10^3^ copies/μL for MS2 (Fig. 2). The results were consistent across five repetitions.

### Standard curves of the quadruple RT-qPCR assay

**Fig 3:**
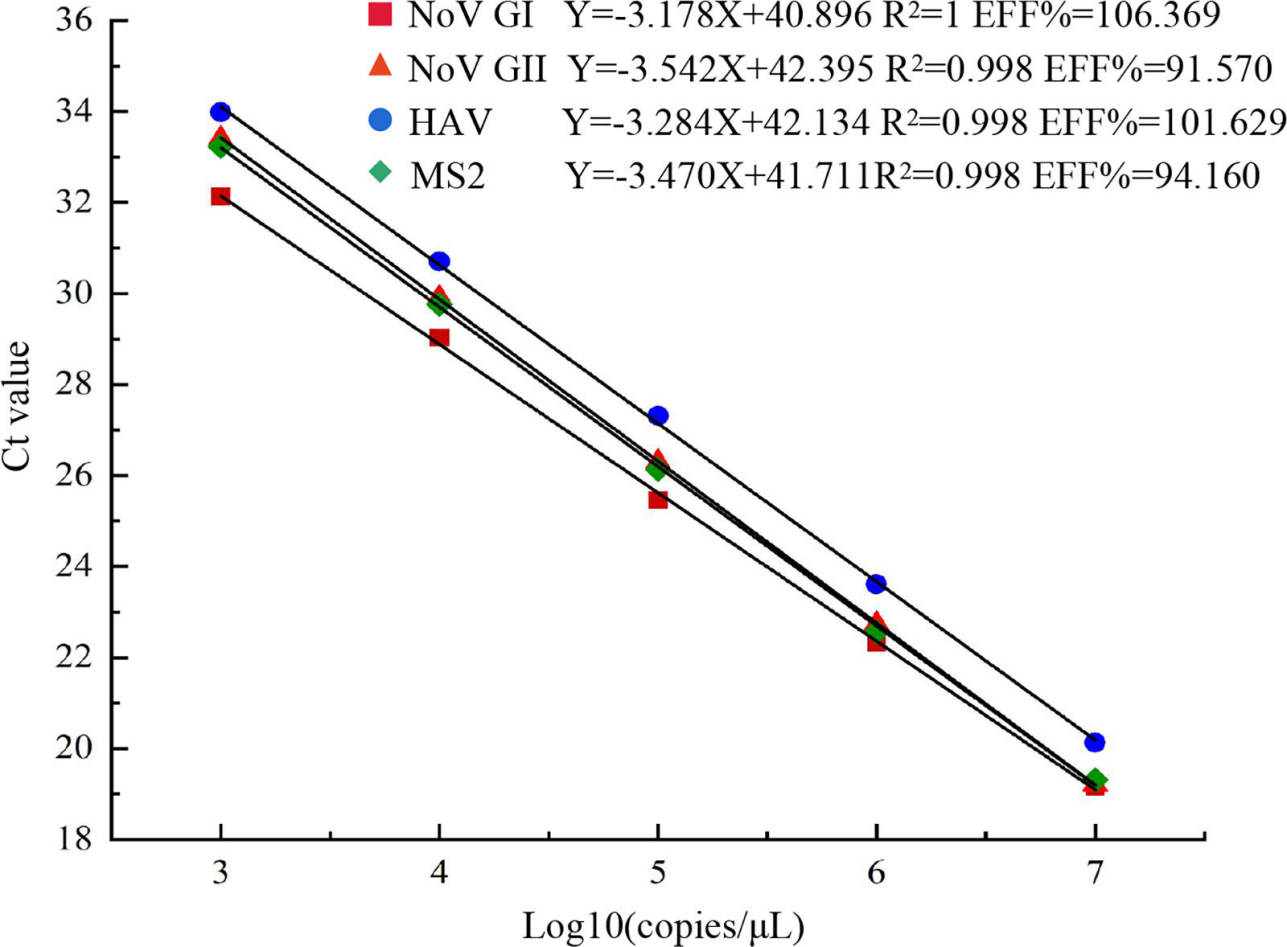
Standard curves of NoV GI, NoV GII, HAV, and MS2 in the quadruple RT-qPCR assay.

Ten-fold serial dilutions of *in vitro* transcribed RNA of NoV GI, NoV GII, HAV, and MS2 were used as templates, and the established quadruple RT-qPCR assay was used to generate standard curves. The results showed that NoV GI, NoV GII, HAV, and MS2 had good linear relationships between Ct values and RNA concentrations within the range of 10^7^ to 10^3^ copies (Fig. 3). The correlation coefficients (R^2^) were 1.000, 0.998, 0.998, and 0.998, respectively, and the amplification efficiencies (EFF%) were 106.37%, 91.57%, 101.63%, and 94.16%, respectively.

### Analysis of the reproducibility

*In vitro* transcribed RNA at concentrations of 10^7^, 10^5^, and 10^3^ copies/μL were used as templates, with three replicates for each concentration, and then subjected to quadruple RT-qPCR amplification. The reproducibility results are shown in Table 2. Analysis of the amplification results showed intra-assay CVs (coefficients of variation) ranging from 0.16% to 1.21%, and inter-assay CVs ranging from 0.55% to 2.11%, with all CVs being less than 5%, indicating good reproducibility of the established quadruple RT-qPCR assay.

**TABLE 2:** Reproducibility results of the quadruple RT-qPCR assay

Virus	*In vitro* transcribed RNA concentration (copies/μL)	Coefficient of variation within groups		Coefficient of variation between groups	
		Ct (x ± s)	CV/%	Ct (x ± s)	CV/%
NoV GI	10^7^	20.70 ± 0.15	0.71	20.69 ± 0.12	0.58
	10^5^	28.42 ± 0.05	0.16	28.19 ± 0.38	1.36
	10^3^	33.91 ± 0.09	0.26	33.70 ± 0.19	0.55
NoV GII	10^7^	21.38 ± 0.13	0.60	21.57 ± 0.39	1.82
	10^5^	29.09 ± 0.75	0.26	28.91 ± 0.23	0.80
	10^3^	35.24 ± 0.10	0.30	34.98 ± 0.32	0.90
HAV	10^7^	22.19 ± 0.21	0.93	22.28 ± 0.13	0.57
	10^5^	29.65 ± 0.15	0.51	29.40 ± 0.22	0.75
	10^3^	35.08 ± 0.42	1.21	34.84 ± 0.74	2.11
MS2	10^7^	21.01 ± 0.11	0.51	20.77 ± 0.23	1.09
	10^5^	28.44 ± 0.20	0.72	28.66 ± 0.20	0.69
	10^3^	33.27 ± 0.31	0.94	34.06 ± 0.70	2.05

### Comparison of pretreatment methods

**Fig 4:**
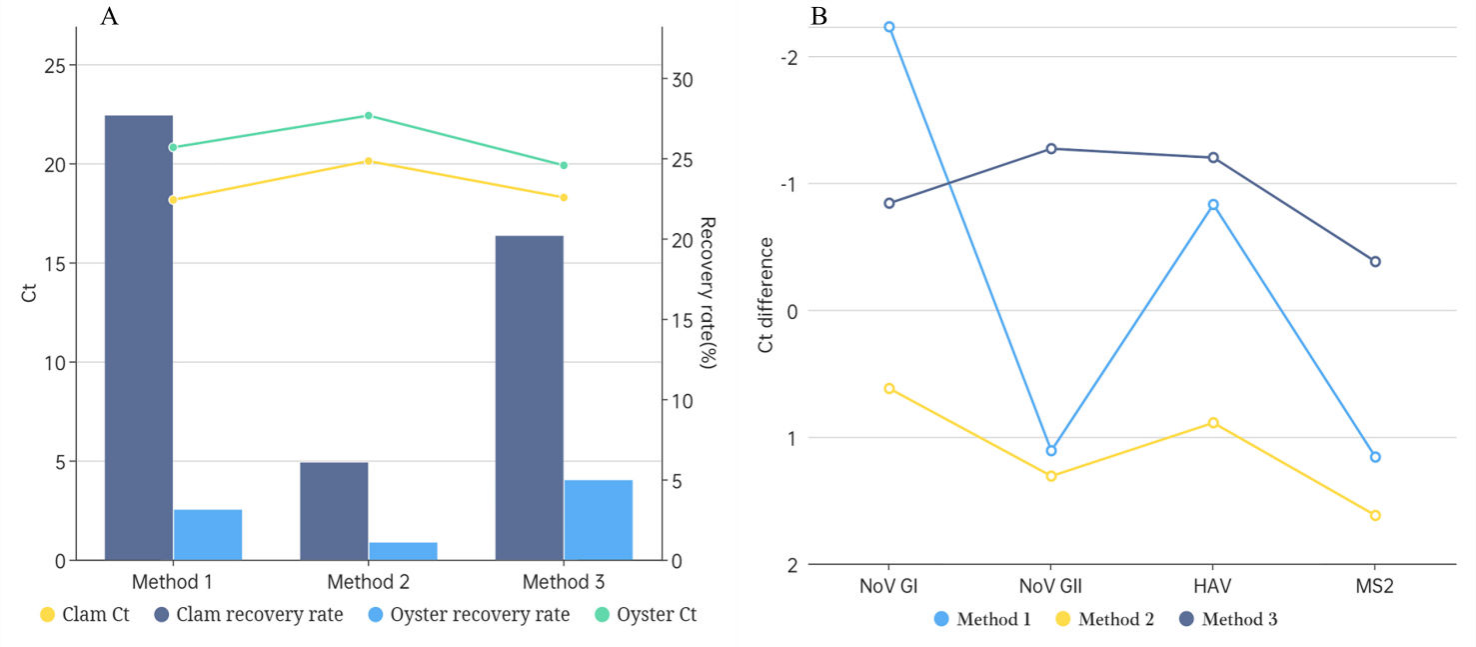
Comparison results of the Ct values, recovery rates, and inhibition indices in three different pretreatment methods. (A) The line graphs represent the Ct values of MS2, and the bar graphs represent the recovery rates of MS2 in the clams and oysters using three different pretreatment methods. (B) The line graphs represent the inhibition indices calculated for NoV GI, NoV GII, HAV, and MS2 in the clams and MS2 Ct values for the oyster group using three different pretreatment methods.

Figure 4 shows the comparison results of recovery rates and inhibition indices for the three virus concentration methods. After treating oyster and clam samples with Method 1, Method 2, and Method 3, the comparison of recovery rates showed that both the clam and oyster groups had better results with Method 1 and Method 3 than with Method 2. The recovery rates obtained by different treatment methods all met the homogeneity of variance, and the differences among the three methods were statistically significant (*P* < 0.05). The inhibition indices of NoV GI, NoV GII, HAV, and MS2 show that both Method 1 and Method 3 are better than Method 2. However, only for NoV GI is the inhibition indices of Method 1 better than that of Method 3, and for the rest of the viruses, Method 3 is better and more stable. The amplified Ct values obtained from the different treatments for the clam and oyster groups conformed to the homogeneity of variance, and the difference between Method 1 and Method 3 was not significant (*P* > 0.05), while the difference between Method 2 and Methods 1 and 3 was significant (*P* < 0.05). Method 1 and Method 3 were better than Method 2 in both clam and oyster groups, but Method 3 had lower Ct values than Method 1.

**Fig 5:**
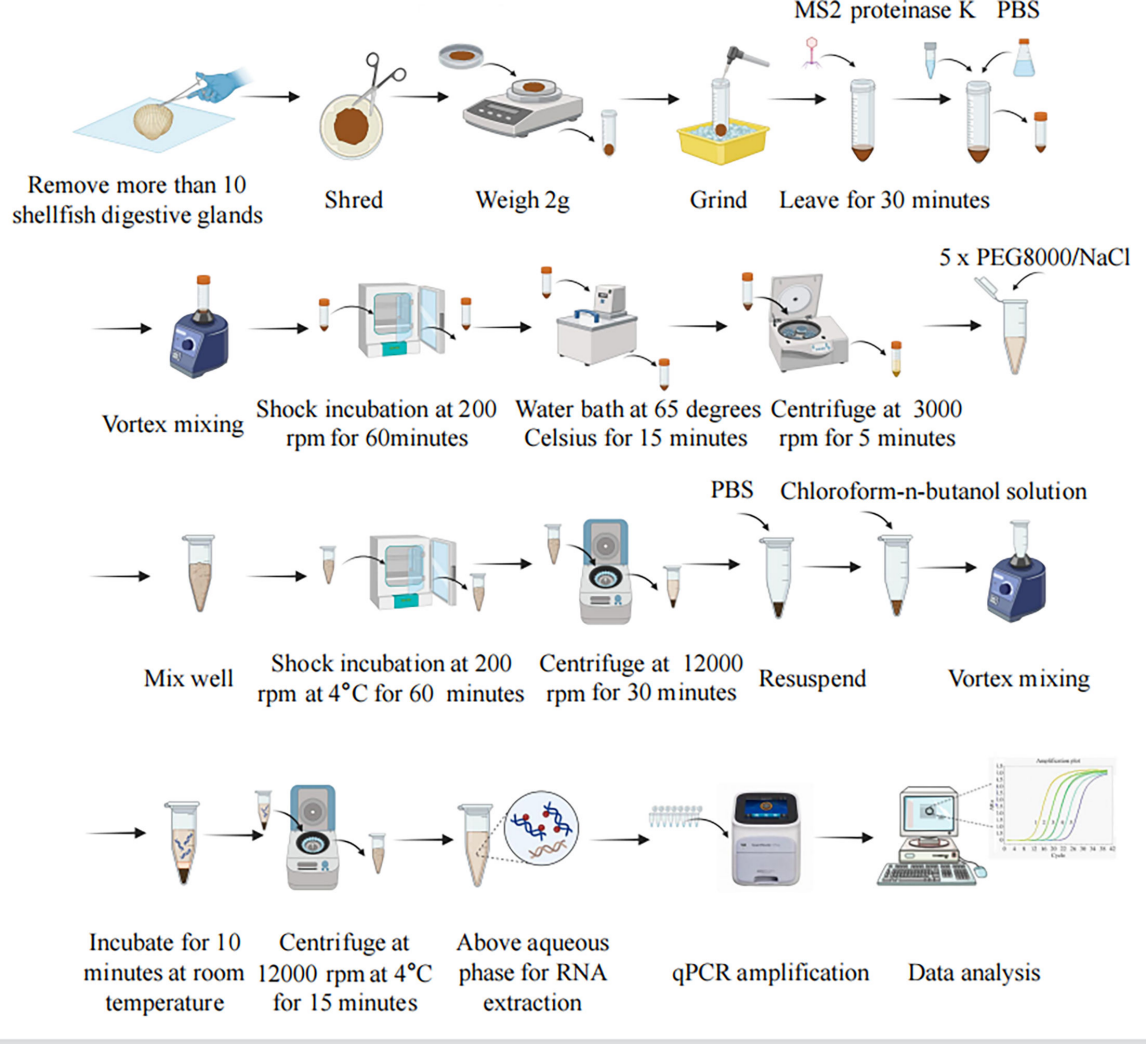
Diagram of the NoV GI, NoV GII, and HAV testing process in shellfish.

By comprehensively comparing the inhibition indices, Ct values of MS2 bacteriophage, and recovery rates of the three different pretreatment methods, it was found that the proteinase K-PEG 8000 precipitation-chloroform extraction method (Method 3) combined with the double viral liquid nucleic acid extraction method was the most stable and effective for the enrichment and extraction of bivalve viruses. Therefore, the proteinase K-PEG 8000 precipitation-chloroform extraction method combined with the double virus liquid nucleic acid extraction method was chosen as the enrichment method for the subsequent experiments. The complete procedure of the bivalve samples is shown in Fig. 5.

### Detection of food-borne viruses in bivalve shellfish products from Hebei province

Using the established quadruple RT-qPCR assay, 337 bivalve shellfish samples from different regions of Hebei Province were tested. The results are shown in Table 3. Among the 337 bivalve shellfish products, 66 samples tested positive for food-borne viruses, with an overall detection rate of 34.12% (115/337). Specifically, the detection rate for NoV GI was 19.88% (67/337), for NoV GII was 20.47% (69/337), and for HAV was 4.75% (16/337). Further analysis showed a mixed infection rate of 11.57% (39/337) for different types of food-borne viruses, primarily NoV GI + GII mixed infections (10.68%, 36/337). In addition, NoV GI + HAV was detected in three samples (0.89%, 3/337). There were statistically significant differences in detection rates among different types of bivalve shellfish samples (*P* < 0.05).

**TABLE 3:** Detection results of NoV GI, NoV GII, and HAV in different bivalve shellfish^*a*^

Sample types	Sample number	Pathogen types					Total positive samples	Positive rate (%)
		NoV GI	NoV GII	HAV	NoV GI+ NoV GII	NoV GI+ HAV		
Clam	116	28	25	8	12	3	47	40.52
Oyster	82	11	16	5	7	0	25	30.49
Scallop	66	8	9	0	4	0	13	19.70
Razor clam	49	17	11	1	10	0	20	40.82
Blood clam	21	3	8	1	3	0	9	42.86
Mussel	3	0	0	1	0	0	1	33.33
Total	337	67	69	16	36	3	115	34.12

^*a*^ χ2 = 14.127, *P* < 0.05.

To compare the detection results of the single and quadruple RT-qPCR assay, 200 bivalve shellfish samples were randomly selected and simultaneously tested using both methods. The results are shown in Table 4. Using the quadruple RT-qPCR assay established in this study, 55 samples tested positive for NoV GI, with a detection rate of 27.5%, 59 samples tested positive for NoV GII, with a detection rate of 29.5%, and 1 sample tested positive for HAV, with a detection rate of 0.5% Using the single RT-qPCR assays, 48 samples tested positive for NoV GI, with a detection rate of 24%, 58 samples tested positive for NoV GII, with a detection rate of 29%, and 2 samples tested positive for HAV, with a detection rate of 1% The total, positive and negative coincidence rates of the quadruple RT-qPCR assay with the single RT-qPCR assays were 98.3%, 99.1% and 98.2%, respectively. No significant difference was noted between the quadruple RT-qPCR assay and the single RT-qPCR assay (*P* = 0.27). Furthermore, the quadruple RT-qPCR assay and the single RT-qPCR assays were significantly in agreement (kappa = 0.945 at 95% CI). The above-mentioned data show that the developed quadruple RT-qPCR assay had a similar diagnostic performance with the single RT-qPCR assays on clinical samples. The recovery rates in this experiment were all greater than 1%, and the inhibition indices were all less than 2.

**TABLE 4:** Comparison of the detection results between the single and quadruple RT-qPCR assays

Pathogen types	Detection methods	Sample number	Positive samples	Positive rate (%)
NoV GI	Monoplex	200	48	24.0
	Multiplex		55	27.5
NoV GII	Monoplex		58	29.0
	Multiplex		59	29.5
HAV	Monoplex		2	1.0
	Multiplex		1	0.5

## DISCUSSION

Food-borne viruses such as NoV and HAV are highly infectious, and once they contaminate water and food, they can easily cause outbreaks or epidemics. During 2019–2020, France experienced emergency visits due to large-scale simultaneous outbreaks of acute gastroenteritis, suspected to be related to the consumption of raw bivalve shellfish (27). In 2016, a study in Guangdong Province reported NoV detection in 75 fecal samples from acute gastroenteritis cases, 331 bivalve shellfish products, and 110 aquaculture water samples, all of which had consistent NoV genotypes, with NoV strains showing high sequence similarity ranging from 95.80% to 99.70% (28). Therefore, the establishment of rapid and suitable detection methods for large-scale detection of food-borne viruses is essential for the protection of public health. The quadruple RT-qPCR assay uses multiple pairs of primers and probes specific for different target species in the reaction system, and by labeling the probes with different fluorescent groups, multiple assays can be achieved in a single amplification reaction, which is a good choice for the above-mentioned situation (29). However, the reaction system of the quadruple RT-qPCR assay is complex, and mismatches between primers and templates can lead to false positives or reduced sensitivity. Previous studies have mainly optimized primer and probe concentrations and annealing temperatures, without considering the impact of reaction component concentrations on multiplex system amplification efficiency. The quadruple RT-qPCR system consumes many more reaction components than the corresponding single RT-qPCR assay because multiple pairs of primers and probes are used to amplify the target sequences. In addition to optimizing primer concentrations and annealing temperatures, this study also adjusted the concentrations of dNTP, Taq Hot-Start DNA polymerase, and Mg^2+^ in the reaction system to determine the optimal reaction system for improving the amplification efficiency of the quadruple RT-qPCR assay. It can be seen that the developed quadruple RT-qPCR method has a similar detection performance as single RT-PCR in Table 5. These findings indicate that quadruple RT-qPCR assay is highly practical. Compared to the traditional single RT-qPCR method, it effectively reduces detection costs and shortens the detection time to 1 hour and 50 minutes, providing strong technical support for large-scale sample testing.

**TABLE 5:** Single and quadruple RT-qPCR assay detection results of NoV GI, NoV GII, and HAV partially RNA-positive samples

Number	Sample types	Sources	Single RT-qPCR assay (cycle threshold, Ct)			Quadruple RT-qPCR assay (cycle threshold, Ct)		
			NoV GI	NoV GII	HAV	NoV GI	NoV GII	HAV
1	Clam	Chengde	35.12			34.51		
2	Clam	Chengde	36.23			34.54		
3	Clam	Zhangjiakou		35.18			33.59	
4	Clam	Zhangjiakou		35.35			35.27	
5	Clam	Chengde	36.21			34.53		
6	Clam	Chengde	35.25			33.12		
7	Oyster	Cangzhou		34.20			34.56	
8	Oyster	Hengshui	36.50			34.24		
9	Oyster	Hengshui	35.51	34.21		35.00	36.10	
10	Razor clam	Zhangjiakou	33.15	33.65		35.32	35.15	
11	Razor clam	Zhangjiakou	36.61	35.25		37.13	35.15	
12	Razor clam	Shijiazhuang	34.58			34.29		
13	Razor clam	Shijiazhuang		35.23			36.10	
14	Scallop	Chengde		33.15			33.26	
15	Scallop	Chengde		35.58			34.40	
16	Scallop	Cangzhou	36.36			36.28		
17	Scallop	Cangzhou	33.19			33.31		
18	Blood clam	Zhangjiakou		36.25			34.54	
19	Blood clam	Zhangjiakou	36.10	34.21		33.33	33.10	
20	Blood clam	Qinhuangdao			35.25			34.54

Bivalve shellfish samples contain large amounts of PCR inhibitors such as polysaccharides, lipids, and proteins, and have lower virus loads compared to other samples, making target gene amplification challenging. Therefore, effective nucleic acid extraction from bivalve shellfish samples is crucial. This study optimized commonly used methods for virus concentration in bivalve shellfish samples and found that Methods 1 and 3 outperformed Method 2 in recovery rate, MS2 amplification Ct values, and inhibition indices. The similar performance of Methods 1 and 3 is inconsistent with other research findings (30), possibly due to variations in supernatant volumes obtained from individual differences in bivalve shellfish samples and handling procedures. Method 3’s better performance compared to Method 2 may be attributed to PEG’s strong water absorption, protein precipitation, and aggregation effects (31). Although studies have shown that Trizol-based RNA extraction methods can effectively extract viral RNA (32), the combination of Trizol lysis-chloroform extraction with the proteinase K method did not yield ideal results in this study. This may be due to the damage to virus particles after proteinase K treatment, making viral RNA more susceptible to degradation by endogenous RNases after Trizol lysis (33). Although both methods 1 and 3 were effective, the Protease K-PEG 8000 precipitation-chloroform method and enrichment extraction of viral nucleic acids from double volumes of viral solution were more stable, with an average recovery of 10.18%, which was effective in improving amplification efficiency. Other bivalve pretreatment methods have been described in a number of publications, including acid adsorption elution (34), direct elution with glycine buffer (35), immunomagnetic bead extraction (36, 37), virus precipitation using Cat-Floc (38) or PEG (34), and solvent extraction using chloroform (39, 40) or chloroform/butanol (41) for virus precipitation, most of which are time-consuming. The Proteinase K-PEG 8000 method proposed by Zhou et al. (42) although less time-consuming and more effective than the above methods, with average recoveries ranging from 9.2% to 9.7 %, still took 1 hour longer than the method optimized in this study. This study also found that recovery rates differed significantly between clams and oysters when using the same enrichment method, which may be related to differences in nutritional content (proteins, fats, and various minerals) and the effectiveness of different methods in removing impurities from different types of bivalve shellfish.

On the other hand, bivalve shellfish are highly valued for their unique flavor and nutritional value, making them an important marine food resource. However, as filter feeders, bivalve shellfish can accumulate food-borne viruses from polluted environments. Outbreaks of food-borne illnesses caused by consuming undercooked bivalve shellfish have been widely reported. In December 2019, the French Public Health Agency reported 43 suspected food-borne outbreaks related to bivalve shellfish, primarily from undercooked or lightly cooked oysters, leading to the closure of 31 aquaculture farms due to NoV contamination (27). In 2016, an outbreak of HAV in the United States was linked to consuming undercooked imported scallops (10), highlighting the risk of HAV transmission through undercooked food. In 2015, approximately 6.20% of food-borne illness cases in Jiangsu Province, China, were associated with the consumption of raw or improperly processed bivalve shellfish (43). In February 2016, a cluster of gastroenteritis cases with symptoms of vomiting and diarrhea occurred in Siming District, Xiamen, China, among a tourist group. Epidemiological investigation and laboratory testing confirmed the cause as NoV from oysters (44). Therefore, this study collected 337 bivalve shellfish samples from Hebei Province between May and December 2023 and applied the established method for actual sample detection, resulting in an overall detection rate of 34.12%. Specifically, NoV GI was detected in 19.88% (67/337), NoV GII in 20.47% (69/337), and HAV in 4.75% (16/337) of the samples. Mixed contamination of different types of food-borne viruses in bivalve shellfish was observed, with a mixed detection rate of 11.57% (39/337), which is consistent with findings from earlier studies (45). This indicates that food-borne virus contamination is still present in bivalve shellfish seafood sold in the market. Previous studies have shown (46) that contamination of bivalve shellfish with food-borne viruses is often related to various environmental factors, including heavy rainfall before harvesting, causing sewage treatment plants to overflow and resulting in fecal contamination of water bodies. Understanding the causes of common contamination in bivalve shellfish production areas, especially precipitation, sewage treatment plant overflow, and fecal contamination of water sources, can help better control food-borne virus contamination in bivalve shellfish. In the future, more samples will be collected and tested over a long period to accurately understand the contamination status of NoV and HAV.

Unlike the ISO standard, this study used MS2 bacteriophage instead of Mengo as the process control virus. MS2 bacteriophage is non-pathogenic to humans and its genome does not contain the target gene sequences of common food-borne viruses, so it can mimic the viral particles to be tested and monitor virus concentration, RNA extraction, and PCR amplification. Blaise-Boisseau et al. (47) used MS2 bacteriophage in the process of QC for the qRT-PCR detection of HAV in food and water and obtained satisfactory results. Rolfe et al. (48) reported the use of MS2 bacteriophage as a process control for the detection of NoV in faecal samples, which was shown to be a simple and reliable method to monitor false-negative PCR results.

Even though the approach in the current study revealed comparative advantages, numerous flaws remained. Although NoV GI and NoV GII are genotypes with high positivity rates in NoV, the detection of other genotypes is also of interest, and in this study, the method was established only for NoV GI and GII. In addition, the enrichment, concentration, and extraction of viruses in bivalve shellfish samples are still the key to the detection of bivalve shellfish samples, and the optimization should be continued in the future, expecting to achieve better results.

### Conclusion

The quadruple RT-qPCR assay developed in this study is highly specific, sensitive, and practically applicable. It is suitable for the rapid and simultaneous detection of NoV GI, NoV GII, and HAV in bivalve shellfish and the related epidemiological investigations, providing an excellent technical tool for the control of the above food-borne virus-related food contaminations.
